# Tick Importin-α Is Implicated in the Interactome and Regulome of the Cofactor Subolesin

**DOI:** 10.3390/pathogens10040457

**Published:** 2021-04-11

**Authors:** Sara Artigas-Jerónimo, Margarita Villar, Alejandro Cabezas-Cruz, Grégory Caignard, Damien Vitour, Jennifer Richardson, Sandrine Lacour, Houssam Attoui, Lesley Bell-Sakyi, Eleonore Allain, Ard M. Nijhof, Nina Militzer, Sophia Pinecki Socias, José de la Fuente

**Affiliations:** 1 SaBio, Instituto de Investigación en Recursos Cinegéticos IREC-CSIC-UCLM-JCCM, Ronda de Toledo s/n, 13005 Ciudad Real, Spain; sartigasjeronimo@gmail.com (S.A.-J.); margaritam.villar@uclm.es (M.V.); 2 Biochemistry Section, Faculty of Science and Chemical Technologies, and Regional Centre for Biomedical Research (CRIB), University of Castilla-La Mancha, 13071 Ciudad Real, Spain; 3 Anses, INRAE, Ecole Nationale Vétérinaire d’Alfort, UMR BIPAR, Laboratoire de Santé Animale, F-94700 Maisons-Alfort, France; alejandro.cabezas@vet-alfort.fr; 4 UMR 1161 Virologie, Laboratoire de Santé Animale, ANSES, INRAE, Ecole Nationale Vétérinaire d’Alfort, Paris-Est Sup, 94700 Maisons-Alfort, France; gregory.caignard@vet-alfort.fr (G.C.); damien.vitour@vet-alfort.fr (D.V.); jennifer.richardson@vet-alfort.fr (J.R.); sandrine.lacour@anses.fr (S.L.); houssam.attoui@vet-alfort.fr (H.A.); eleonore.allain@hotmail.fr (E.A.); 5 Tick Cell Biobank, Institute of Infection, Veterinary and Ecological Sciences, University of Liverpool, 146 Brownlow Hill, Liverpool L3 5RF, UK; l.bell-sakyi@liverpool.ac.uk; 6 Institute for Parasitology and Tropical Veterinary Medicine, Freie Universität Berlin, 14163 Berlin, Germany; ardmenzo.nijhof@fu-berlin.de (A.M.N.); nina.militzer@fu-berlin.de (N.M.); sophia.Pinecki@fu-berlin.de (S.P.S.); 7 Center for Veterinary Health Sciences, Department of Veterinary Pathobiology, Oklahoma State University, Stillwater, OK 74078, USA

**Keywords:** subolesin, interactome, regulome, importin, histone, epigenetic, tick, akirin

## Abstract

Ticks and tick-borne diseases (TBDs) represent a burden for human and animal health worldwide. Currently, vaccines constitute the safest and most effective approach to control ticks and TBDs. Subolesin (SUB) has been identified as a vaccine antigen for the control of tick infestations and pathogen infection and transmission. The characterization of the molecular function of SUB and the identification of tick proteins interacting with SUB may provide the basis for the discovery of novel antigens and for the rational design of novel anti-tick vaccines. In the present study, we used the yeast two-hybrid system (Y2H) as an unbiased approach to identify tick SUB-interacting proteins in an *Ixodes ricinus* cDNA library, and studied the possible role of SUB as a chromatin remodeler through direct interaction with histones. The Y2H screening identified Importin-α as a potential SUB-interacting protein, which was confirmed in vitro in a protein pull-down assay. The *sub* gene expression levels in tick midgut and fat body were significantly higher in unfed than fed female ticks, however, the *importin-α* expression levels did not vary between unfed and fed ticks but tended to be higher in the ovary when compared to those in other organs. The effect of *importin-α* RNAi was characterized in *I. ricinus* under artificial feeding conditions. Both *sub* and *importin-α* gene knockdown was observed in all tick tissues and, while tick weight was significantly lower in *sub* RNAi-treated ticks than in controls, *importin-α* RNAi did not affect tick feeding or oviposition, suggesting that SUB is able to exert its function in the absence of Importin-α. Furthermore, SUB was shown to physically interact with histone 4, which was corroborated by protein pull-down and western blot analysis. These results confirm that by interacting with numerous tick proteins, SUB is a key cofactor of the tick interactome and regulome. Further studies are needed to elucidate the nature of the SUB-Importin-α interaction and the biological processes and functional implications that this interaction may have.

## 1. Introduction

Ticks are obligate blood-feeding arthropod vectors of pathogenic viruses, bacteria, protozoa and helminths, and are responsible for highly prevalent tick-borne diseases (TBDs) worldwide [1]. Ticks are the second most common arthropod, after mosquitos, that transmit pathogens to humans and the most important vector in domestic animals [2]. This situation imposes a real burden for human and animal health, which is increasing day by day [1]. Nowadays, different strategies have been developed to control ticks and TBDs by reducing tick infestations and pathogen transmission. Some examples of these methods are the traditional use of chemical acaricides, which are generally environmental contaminants and can lead to acaricide-resistant ticks. Botanical acaricides, management of habitat and personal preventive control measures, among others, are more environmentally friendly methods but with a low success rate in the control of TBDs [3,4,5,6,7,8,9]. Vaccines constitute the safest and more effective approach to control ticks and TBDs [10], but new antigens or improved vaccine formulations are required to advance development of control interventions. Although vaccine efficacy against arthropods has been described [11,12,13,14,15,16], the combination of different protective antigens such as interacting proteins (involved in interactome or functional and physical protein–protein interactions) may advance research in this area [17,18].

Subolesin (SUB) is a tick protein that was first reported in 2003 as the protective antigen derived from the 4D8 clone in *Ixodes scapularis* [19]. Subolesin and its functional ortholog in vertebrates, Akirin (AKR), are regulatory cofactors conserved throughout metazoan evolution without catalytic or DNA-binding capability [20,21]. SUB/AKR acts in concert with Relish/Toll-like receptor (TLR)-nuclear factor kappa-light-chain-enhancer of activated B cells (NF-kB) inducing immune deficiency (IMD) and tumor necrosis factor (TNF)/TLR signaling pathways that are involved in the immune response to pathogen infection in ticks and in vertebrate organisms [21,22,23,24,25,26]. Furthermore, SUB is highly conserved at gene and protein levels in the Ixodidae with a protective capacity as a vaccine antigen for the control of tick infestations and pathogen infection/transmission [2,21].

Study of the tick interactome contributes to the understanding of cellular processes and biological functions and can lead to the discovery of drug and vaccine targets for disease prevention and treatment [17,21,27]. Thus, understanding the interactome of a protein such as the transcription cofactor SUB is important to unravel its implications in gene regulation and host–pathogen interactions. In a previous study, two SUB-interacting proteins were identified, GI and GII, through which SUB may exert its function in the regulation of gene expression, showing that the effect of gene knockdown was similar for GII and SUB [22]. However, how SUB functions in the tick regulome (transcription factors/cofactors targeting genes interactions) is still unknown. Recently, human AKR2 was suggested to function as a chromatin remodeler interacting physically with histone H3.1, thus providing new information on the interactome and regulome of this protein with a novel role in epigenetic regulation [28].

To provide deeper understanding of the SUB interactome and regulome, in the present study we focused on the identification of tick SUB-interacting proteins and studied the possible role of SUB as a chromatin remodeler through direct interaction with histones. The results identified Importin-α as a SUB-interacting protein with possible implications for signal transduction and gene regulation.

## 2. Results and Discussion

### 2.1. Importin-α Is Part of the SUB Interactome with a Putative Function in Translocation to the Cell Nucleus

**Figure 1:**
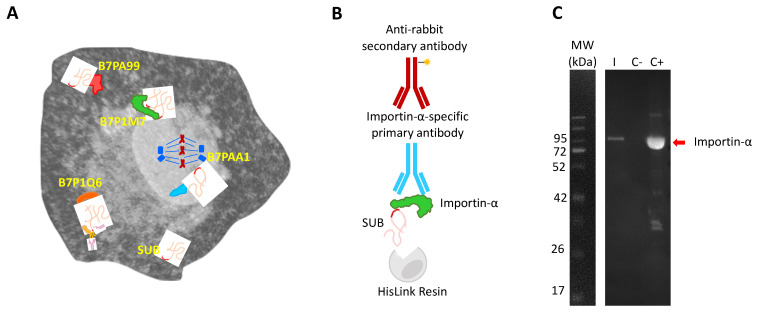
Characterization of the tick SUB interactome. (**A**) Graphical representation of the results of the Y2H screening in tick cells by which Importin-α (B7P1M7; green) together with other tick proteins (B7PA99, B7PAA1 and B7P1Q6) were identified as interacting with SUB (pink and red). (**B**) Graphical representation of the components of the protein pull-down experiment using a resin with a high level of tetradentate-chelated nickel, which captures polyhistidine-tagged proteins. (**C**) Western blot analysis of the SUB-Importin-α interaction. SUB was incubated with Importin-α and recovered using the HisLink resin through the SUB His-tag (I). Importin-α was incubated only with the resin as negative control (C-) and recombinant importin-α was included as a positive control (C+). Specific primary and secondary antibodies were used to detect the presence of Importin-α (84 kDa; red arrow). The molecular weight of Importin-α is 57 kDa, but the recombinant protein used in this experiment contains a GST tag used for its purification.

The SUB/AKR proteins have been described as protective antigens of ticks and other arthropods, making them interesting candidates for the vaccines targeting arthropods [21]. Furthermore, SUB and its vertebrate ortholog, AKR2, act as cofactor regulatory proteins that do not directly interact with DNA, and thus exert their function through interactions with other proteins [21]. To characterize the tick SUB interactome, the identification of SUB-interacting proteins was performed, and four proteins were identified with different confidence scores (depending on the number of identified clones for each protein) [18] (Figure 1A). Three of them were identified with a confidence B score (two clones each); that is, B7PA99 (uncharacterized protein), B7PAA1 (transforming acidic coiled-coil containing protein) and B7P1Q6 (TRAF-type domain-containing protein). The fourth protein, B7P1M7 (Importin-α, also known as Karyopherin-α), was identified with a confidence score of A (three clones). B7PA99 is an uncharacterized protein with 76% sequence identity to an *Ixodes scapularis* transforming acidic coiled-coil-containing protein 3 isoform X4] (XP_029836069.1) and there is little information related to the role of this protein in the tick interactome. However, the B7PAA1 transforming acidic coiled-coil containing protein has been implicated in mitotic spindle organization (https://www.uniprot.org/uniprot/B7PAA1, accessed on 12 October 2020) and it is linked to different types of cancers in humans [29,30,31,32]. The B7P1Q6 TRAF-type domain-containing protein has been shown to be involved in innate immunity of *Haemaphysalis longicornis* ticks against bacterial infection [33], suggesting a functional interaction with SUB due to its role in the innate immune response [34,35].

Due to its highest confidence score in SUB–protein interactions and the possible role in SUB transport into the nucleus, we then focused on Importin-α, a protein family conserved in all metazoans. The SUB-Importin-α interactions were corroborated by in vitro protein pull-down and Western blot analysis with Importin α-specific primary antibodies (Figure 1B,C). Importin-α family members act as a linker between the nuclear localization sequence (NLS) cargo proteins, while members of the Importin-β family facilitate protein translocation into the nucleus through nuclear pores [36,37]. SUB bears two distinct NLS domains, thus representing a good candidate for Importin-α-mediated transport into the nucleus [21]. Although only a single Importin-α is encoded in ticks, the *importin-α* gene family has undergone broad expansion during eukaryotic evolution. In humans, the genome contains several genes coding for Importin-α [36].

**Figure 2:**
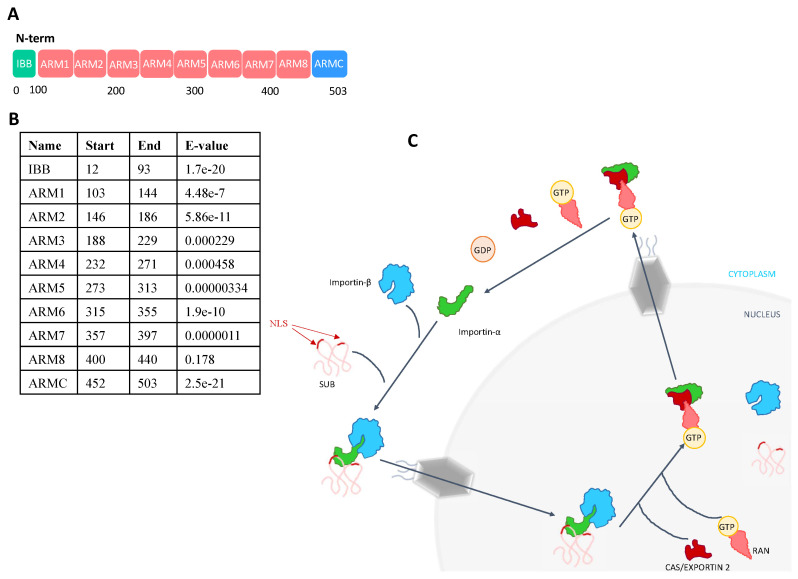
Protein domain architecture analysis and transport cycle of Importin-α. (**A**) Graphical representation of the predicted protein domains of tick Importin-α. These include an Importin-β binding domain (IBB), eight Armadillo/beta-catenin-like repeats (ARM 1-8) and an atypical ARM repeat in the C-terminal region (ARMC). (**B**) Confidence (E-value) of the predicted tick Importin-α domains and repeats. The analysis and the prediction were carried out using the SMART tool (http://smart.embl-heidelberg.de/, accessed on 12 October 2020). (**C**) Importin-α binds to NLS-cargo proteins such as SUB and also to Importin-β. Importin-β/α-SUB could be translocated into the nucleus by the interaction with the nuclear pore complexes. Then, proteins such as Exportin-2 and Ran-GTP trigger the dissociation of the Importin-β/α-SUB complex through binding to Importin-α. Finally, Importin-α is recycled to the cytoplasm.

The *I. scapularis* Importin-α is a protein composed of 521 amino acids and it shares the typical protein domain architecture with Importin-α family members in other species (Figure 2A,B) [37]. It is composed of one Importin-β binding domain (IBB) in the N-terminal region that is involved in the interaction with Importin-β and the formation of the Importin-α/Importin-β-cargo protein ternary complexes (Figure 2C). The Importin-α IBB domain is followed by eight Armadillo/beta-catenin-like repeats (ARM 1-8), which contain approximately 40 amino acids per ARM domain (Figure 2A,B). These ARM repeats constitute the NLS-binding pocket of the Importin [36,37]. In the C-terminal region, an atypical ARM repeat (ARMC) is found, as in other eukaryotic proteins (https://pfam.xfam.org/family/Arm_3, accessed on 12 October 2020) (Figure 2A,B). This region can be targeted by proteins involved in the release of the NLS-cargo protein, which is essential for the regulation of the classical transport mechanism (Figure 2C) [38]. Importin-α family members have a key role in entering the nucleus through the nuclear pores with different NLS cargo proteins such as SUB. Importin-α directly interacts with NLS-proteins thought the ARM repeats in its structure. The N-terminal IBB domain binds to Importin-β and forms a ternary complex as the first step in nuclear transport. However, this IBB domain may also mimic an NLS-protein, thereby autoinhibiting interaction with the NLS-binding region when Importin-α is not bound to Importin-β. This autoinhibitory function is not very strong and NLS-proteins can still interact with Importin-α in the absence of Importin-β [36]. The Importin-α /Importin-β-NLS protein ternary complex is targeted to the nuclear pore complexes (NPCs). This pathway consists of NPCs, nuclear transport receptors (NTRs) and the small GTPase RAN system. NPCs are composed of multiple copies of different proteins called nucleoporins (Nups), grouped according to their sequence motifs, structural folds and functions. Importin-β achieves NPC permeability via binding to the flexible gate (FG) repeats of FG-Nups. Once the Importin-α/Importin-β-NLS protein ternary complex targets the NPC, it is translocated into the nucleus via Importin β activity. Then, the ternary complex is dissolved, and cargo proteins are released through the interaction of nuclear Ras-related nuclear protein (Ran) GTP with Importin-β. Both Importins are then recycled back to the cytoplasm by interaction with other proteins such as RanGTP and CAS/CSE1L [37,39] (Figure 2C). In ticks, this recycler protein or exportin is also present (i.e., *I. scapularis* exportin-2 A0A4D5RQ98) (Figure 2C). It has been described that the C-terminal region of Importin-α can interact directly with the nucleoporin Nup153, involved in the NPCs, thereby promoting the translocation of Importin-α/Importin-β-cargo protein ternary complexes, thus suggesting that beyond its adaptor function Importin-α can act as an active translocation mediator [38].

### 2.2. The SUB-Importin-α Interaction Is Not Essential for SUB Function

Tick SUB can be found either in the cytoplasm or in the nucleus, in relation to its two NLS domains involved in protein transport into the nucleus [21]. While identification of a physical interaction between SUB and Importin-α strongly suggested that SUB might enter the nucleus by the Importin-α pathway, other nuclear entry pathways might come into play. To address this issue, we studied *sub* and *importin-α* gene expression in vivo in *I. ricinus* ticks to gain information on the SUB-Importin-α interaction and its functional implications.

**Figure 3:**
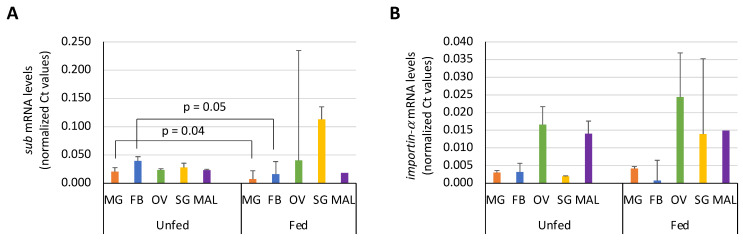
Expression profile of *sub* and *importin-α* in *Ixodes ricinus* organs. The mRNA levels of (**A**) *sub* and (**B**) *importin-α* were determined by qRT-PCR in unfed and fed female *I. ricinus* midgut (MG), fat body (FB), ovary (OV), salivary glands (SG) and Malpighian tubules (MAL). The Ct values were normalized against tick elongation factor (average + S.D.) and compared between fed and unfed ticks by Student’s t-Test (*p* < 0.05; 3 biological replicates with 7–10 ticks per replicate).

First, we studied by qRT-PCR the expression profile of *sub* and *importin-α* in various *I. ricinus* organs including midgut, fat body, ovary, salivary glands and Malpighian tubules of unfed and fed female ticks (Figure 3A,B). The *sub* gene expression levels in midgut and fat body were significantly higher in unfed than in fed female ticks (Figure 3A), which supports a role for this protein in tick feeding [21]. Although without significant differences, *sub* mRNA levels were, as previously reported in *Haemaphysalis flava* [40], higher in the ovaries and salivary glands of fed ticks compared to unfed ticks (Figure 3A). However, the *importin-α* expression levels did not vary between unfed and fed ticks but tended to be higher in the ovaries when compared to those in other organs (Figure 3B). These results concur with the reported role of Importin-α in gametogenesis in *Drosophila melanogaster* and in germ cell development and somatic cell growth in *Caenorhabditis elegans* [36,41].

**Figure 4:**
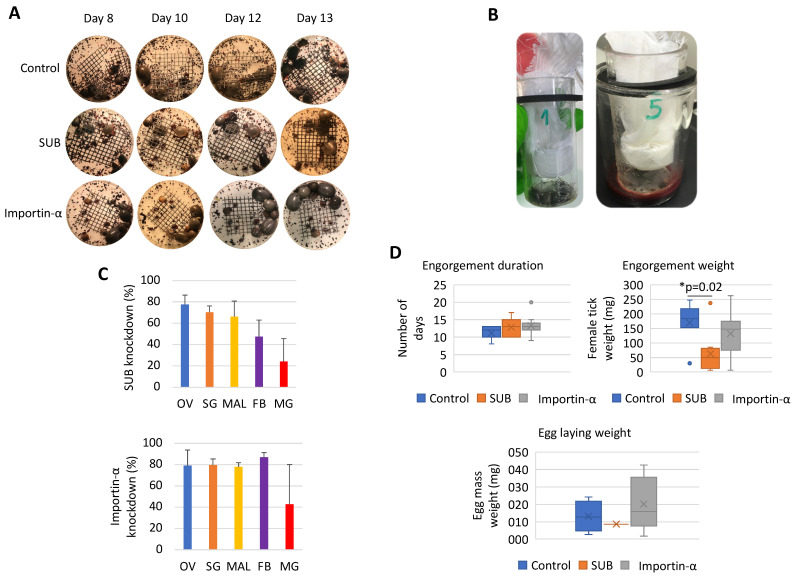
Effect of *sub* and *importin-α* gene knockdown by RNAi in female *Ixodes ricinus* ticks. (**A**) Pictures taken on the inside of the feeding units from the different groups (control GFP, SUB and Importin-α). Differences in tick engorgement, morphology and survival could be observed during feeding. (**B**) Representative pictures of the feeding units with attached ticks on the membrane (left) and during blood feeding (right). (**C**) The *sub* and *importin-α* mRNA levels (Ct-values) were determined by qRT-PCR after RNAi in midgut (MG), fat body (FB), ovary (OV), salivary glands (SG) and Malpighian tubules (MAL) in comparison with control samples. Normalized Ct values were used to calculate knockdown percentages. (**D**) Duration of the feeding period, weight of engorged female ticks and egg batch masses were recorded for each of the treatment groups. Values of *sub* and *importin-α* RNAi groups were compared to controls by one-way ANOVA test with post-hoc Tukey HSD (https://astatsa.com/OneWay_Anova_with_TukeyHSD/, accessed on 12 October 2020; *p* < 0.05; *n* = 80 ticks per group).

Then, the effect of *importin-α* RNAi was characterized in vivo in *I. ricinus* ticks (Figure 4A,D). Ticks were fed under artificial feeding conditions (Figure 4A,B). Both *sub* (23–78%) and *importin-α* (41–87%) gene knockdown was observed in all tick tissues (Figure 4C). As expected from previous results [2,42], tick weight was significantly lower in *sub* RNAi-treated ticks than in controls (Figure 4D). However, *importin-α* RNAi did not affect tick feeding or oviposition (Figure 4D), suggesting that SUB is able to enter the nucleus and exert its function in the absence of Importin-α. This finding suggests that SUB, like AKR, evolved to exert its function through different mechanisms and interaction with other proteins/molecules with functional complementarity.

**Figure 5:**
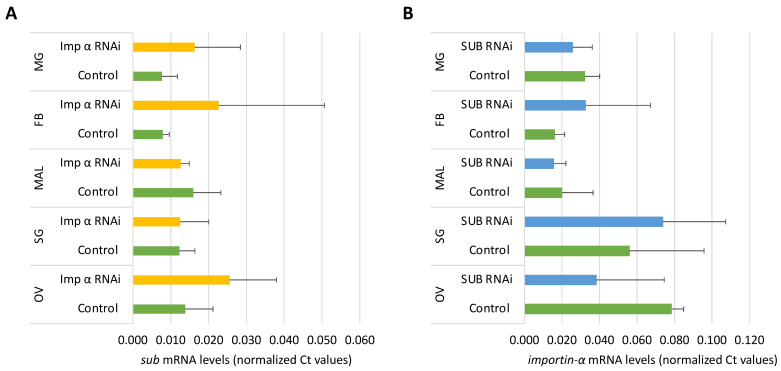
Co-regulation assessment of SUB and Importin-α. (**A**) The *sub* mRNA levels were determined in control and *importin-α* RNAi-treated ticks. (**B**) The *importin-α* mRNA levels were determined in control and *sub* RNAi-treated ticks. The mRNA levels were determined by qRT-PCR in *Ixodes ricinus* midgut (MG), fat body (FB), ovary (OV), salivary glands (SG) and Malpighian tubules (MAL). The Ct values were normalized against tick elongation factor (average + S.D.) and compared between fed and unfed ticks by Student’s t-Test (*p* < 0.05; 3 biological replicates with 7–10 ticks per replicate). No significant differences were obtained.

To further address the hypothesis that SUB enters the nucleus and exerts its function independently of the presence of Importin-α, the *sub* and *importin-α* mRNA levels were assessed after gene knockdown in different tick tissues (Figure 5A,B). The results did not show significant differences between *sub*/*importin-α* and control groups, suggesting that these proteins are not co-regulated (Figure 5A,B). These results further support the hypothesis that SUB may enter the nucleus not only by interaction with Importin-α but also through other unknown mechanisms.

Previously, other functions of Importin-α besides nuclear transport have been described [38]. Among them, Importin-α may be involved in gene regulation in the nucleus under particular stress conditions. It may, moreover, act as a cofactor for transcription factors, conferring enhanced capacity to bind to DNA-specific sequences in yeast [38,43]. In addition, Importin-α has been reported to have a chromatin-related function in the regulation of p21 gene expression in humans [38,44] and in chromatin formation and DNA methylation in *Neurospora crassa* [38,45]. These multiple Importin-α functions in gene regulation and epigenetic mechanisms suggest a novel key role of this protein within the SUB interactome and regulome by both participating in the transport of SUB into the nucleus and acting as a SUB cofactor in the regulation of gene expression.

### 2.3. SUB Interaction with Histone H4

**Figure 6:**
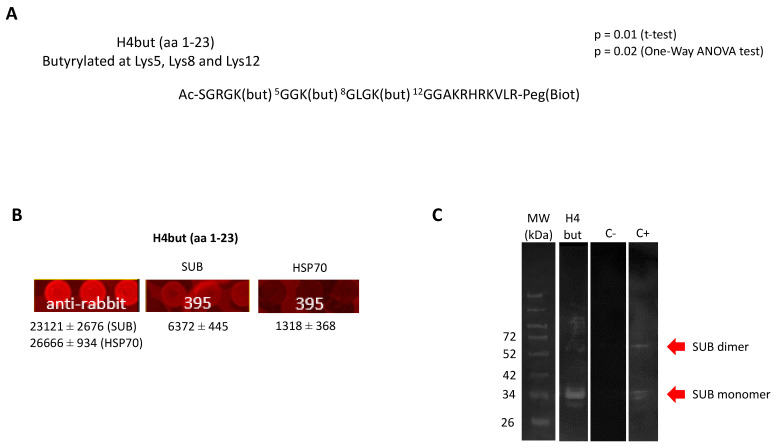
Identification and characterization of the SUB-H4but physical interaction. (**A**) Representation of histone H4 butyrylated at lysines 5, 8 and 12 identified in the histone microarray as a SUB-interacting peptide. (**B**) Graphical representation of the fluorescence from SUB-H4but physical interaction on the histone microarray. Positive (anti-rabbit) and negative (HSP70) controls are shown. Relative fluorescence is shown in RFU (average ± S.D.; 2 biological replicates). (**C**) Corroboration of the interaction of recombinant SUB produced in *E. coli* and H4but in vitro by protein pull-down and Western blot with SUB-specific primary antibodies. Immunoreactive proteins were visualized by chemiluminescence. Streptavidin beads incubated only with SUB and recombinant SUB were included as negative (C-) and positive (C+) controls, respectively.

The functional ortholog of SUB in humans, AKR2, has been suggested to physically interact with the histone H3.1 for chromatin remodeling and regulation of gene expression [28]. This finding raised the question of whether SUB is also involved in chromatin remodeling in ticks. To address this question, we performed a histone peptide array analysis with histone peptides (Data S1). The results identified an H4but peptide (H4 K5,8,12but; amino acids 1–23; Ac-SGRGK(but) 5GGK(but) 8GLGK(but) 12GGAKRHRKVLR-Peg(Biot); butyrylated at lysines 5, 8 and 12) as being involved in a putative SUB-histone interaction (Figure 6A,B).

Although the identified histone peptide was derived from human sequences, the *I. scapularis* histone 4 (Q4PM69) is 100% homologous to the human ortholog [46]. In fact, although *I. scapularis* H1, H2A, H2B and H3 are more closely related to other tick histones, H4 is closely related to mammalian H4 [47]. This interaction was corroborated by protein pull-down and Western blot analysis (Figure 6C). These results documented for the first time the physical interaction of SUB with H4but and suggested a novel mechanism for post-translational modification of tick H4 with new functional implications of SUB in chromatin remodeling and the regulation of gene expression. As described above, in the absence of SUB, Importin-α is unaffected but the tick life cycle is compromised. On the other hand, in the absence of Importin-α, SUB is unaffected, and the tick life cycle is normal. Could SUB be exerting Importin-α’s function related to regulation of gene expression? Further studies are needed to elucidate the nature of this interaction and the biological processes and functional implications that this interaction could have.

## 3. Materials and Methods

### 3.1. SUB Cloning

The SUB-encoding open reading frame (ORF) was amplified by standard PCR (Platinum taq; Invitrogen, Carlsbad, CA, USA) using *sub*-specific primers flanked with the Gateway cloning sites 5′-GGGGACAACTTTGTACAAAAAAGTTGGC and 5′-GGGGACAACTTTGTACAAGAAAGTTGG. PCR products were cloned by in vitro recombination (Gateway System BP cloning reaction; Invitrogen) into pDONR207 (Invitrogen). The *sub* ORF was then transferred from pDONR207 into the Gal4-BD yeast two-hybrid vector pDEST32 according to the manufacturer’s recommendations (LR cloning reaction; Invitrogen).

### 3.2. Yeast Two-Hybrid Screening Procedure

Our yeast two-hybrid (Y2H) protocol was described in detail by Caignard et al. [48]. Briefly, the Y2H gold yeast strain (Clontech Laboratories, Mountain View, CA, USA) was transformed with the pDEST32 plasmid encoding Gal4-DB fused to SUB. After confirming that the *sub* ORF did not induce autonomous transactivation of the *HIS3* reporter gene, screening was performed on synthetic medium lacking histidine ([His-] medium) and supplemented with only 5 mM of 3-amino-1,2,4-triazole (3-AT; Sigma-Aldrich, St. Louis, MI, USA). A mating strategy was used for screening an *Ixodes ricinus* tick cDNA library cloned into the Gal4-AD pDEST22 vector (Invitrogen) and previously established in Y187 yeast strain (Clontech Laboratories) [49]. Yeast cells were plated on a selective medium lacking histidine and supplemented with 5 mM 3-AT to select for interaction-dependent transactivation of the *HIS3* reporter gene. AD-cDNAs from [His+] colonies were amplified by PCR and sequenced to identify the proteins interacting with SUB.

### 3.3. Expression Profile of Sub and Importin-α in I. Ricinus Organs

Unfed and fed adult female *I. ricinus* ticks from a laboratory colony (Freie Universität Berlin, Berlin, Germany) were used for this experiment. For fed female organ dissection, female ticks were fed in vivo for 4 days using rabbits as host until they were partially-fed. The five principal organs (salivary glands, fat body, Malpighian tubules, ovary and midgut) from unfed and fed female *I. ricinus* (3 biological replicates with 7–10 ticks per replicate) were dissected and put into TRIzol Reagent (Invitrogen) for RNA extraction. RNA was extracted using Direct-Zol RNA Miniprep Plus Kit (Zymo Research Europe GmbH, Freiburg im Breisgau, Germany). RNAs were cleared of gDNA and retrotranscribed using iScript gDNA clear cDNA synthesis kit (BioRad, Hercules, CA, USA). cDNAs were used to characterize the expression profile of SUB and Importin-α within different organs of unfed and fed *I. ricinus* females by qPCR using gene-specific oligonucleotide forward (F) and reverse (R) primers (*sub*, F: 5′ CCAAACGGTAGATCGCCCAA 3′, R: 5′ GTCGCATTTCCTCCCGAATG 3′; *Importin-α*, F: 5′ TGGGCGCTCTCCAATCTTTG 3′, R: 5′ TCACTGCCTGGATCTTGTCG 3′), SsoAdvanced Universal SYBR Green Supermix (BioRad) and the CFX96 Touch Real-Time PCR Detection System (BioRad). A dissociation curve was run at the end of the reaction to ensure that only one amplicon was formed, and that the amplicons were denatured consistently in the same temperature range for every sample. The cDNA levels were normalized against tick elongation factor (*elf*, F: 5′ CAAGATTGGTGGTATCGGCA 3′, R: 5′ GACCTCAGTGGTGATGTTGGC 3′) using the genNorm Delta-Delta-Ct (ddCt) method as previously described [50]. Normalized Ct values were compared between fed and unfed ticks by Student’s t-Test (*p* < 0.05).

### 3.4. Corroboration of SUB-Importin-α Interaction by Protein Pull-Down

The synthetic *I. scapularis* histidine-tag recombinant SUB (Genbank accession number AY652654.1) with optimized codon usage for *Escherichia coli* was produced in *E. coli* BL21 and purified to >95% purity by Ni affinity chromatography using 1 mL HisTrap FF columns mounted on an AKTA–FPLC system (GE Healthcare, Piscataway, NJ, USA) in the presence of 7 M urea lysis buffer as previously described [51]. Recombinant SUB and *I. scapularis* recombinant Importin-α fused to glutathione-S-transferase (GST), also produced in *E. coli* (commercially synthesized by GenScript, Piscataway, NJ, USA), were incubated in 100 mM HEPES (pH 7.5) and 10 mM imidazole (binding and wash buffer) for 1 h with shaking, at a concentration ratio of 2:1. HisLink Protein Purification Resin (Promega Corporation, Madison, WI, USA) was washed following the manufacturer’s recommendations and blocked with 1% BSA (Thermo Fisher Scientific, Waltham, MA, USA) for 30 min with shaking. Then, the resin was washed with binding/wash buffer and incubated with SUB-Importin-α interaction (2:1) for 1 h with shaking. For the negative control, the same resin was incubated only with Importin-α to verify that only his-tag SUB was able to link to the resin. After the 1 h incubation, the supernatant was removed, and the resin was washed twice with binding/wash buffer. SUB-Importin-α complexes were eluted from the resin using elution buffer (100 mM HEPES, pH 7.5, 500 mM imidazole). The eluted fractions were recovered and Laemmli sample buffer with β-mercaptoethanol was added for Western blot analysis. Recombinant Importin-α (6 µg) was used as a positive control. Samples were separated by electrophoresis in a 12% sodium dodecyl sulfate (SDS) polyacrylamide precast gel (ClearPage, Cole-Parmer, Vermon Hills, IL, USA) and transferred to a nitrocellulose blotting membrane (GE Healthcare Life Sciences, Pittsburgh, PA, USA). The membrane was blocked with 3% BSA in Tris-buffered saline (TBS; 150 mM NaCl, 50 mM Tris-HCl, pH 7.5) for 2 h at room temperature (RT) and washed four times with TBS-0.05% Tween 20. Human KPNA6-specific primary antibodies (Biorbyt Ltd., Cambridge, UK) were used for Importin-α detection. Antibodies were diluted in TBS and incubated with the membrane overnight at 4 °C. Goat anti-rabbit IgG (whole molecule) peroxidase antibodies (dilution 1:1000; Sigma-Aldrich) diluted in TBS with 3% BSA were used as secondary antibodies and incubated with the membrane for 2 h at RT. The membrane was finally washed five times with TBS-0.05% Tween 20, and immunoreactive proteins were visualized with chemiluminescence by incubating the membrane for 1 min with Pierce ECL Western blotting substrate (Thermo Fisher Scientific).

### 3.5. RNA Extraction and Synthesis of Tick cDNA for dsRNA Preparations

Total RNA was extracted from *I. ricinus* nymphs (*n* = 3–5 nymphs) partially fed using an in vitro feeding procedure with bovine blood [52]. Whole nymph bodies were immersed in TRIzol Reagent (Invitrogen) for RNA extraction. RNA was extracted using Direct-Zol RNA Miniprep Plus Kit (Zymo Research Europe GmbH). RNAs were cleared of genomic DNA and retrotranscribed using a Superscript IV First-Strand Synthesis Kit (Invitrogen). The cDNAs were then used as template for dsRNA synthesis.

### 3.6. Gene Knockdown by RNA Interference in Ticks

Oligonucleotide forward (F) and reverse (R) primers containing T7 promoter sequences (underlined) at the 5′-end to support in vitro transcription and synthesis of dsRNA were synthesized for *I. ricinus sub* and *importin-α* (*sub*, F: 5′-TAATACGACTCACTATAGGCCCAAACGAGCCAGATGTATG-3′, R: 5′-TAATACGACTCACTATAGGGAAGGTGAAGAGGGGCTGGT-3′; *importin-α*, F: 5′-TAATACGACTCACTATAGGCCGGCTGCGCTACAAGAAT-3′, R: 5′-TAATACGACTCACTATAGGTTCCGGTCTCGATCACCTC-3′) and used for *sub* and *importin-α* dsRNA synthesis from *I. ricinus* nymph cDNA using the Phusion™ High-Fidelity DNA Polymerase (Thermo Fisher Scientific) and the T7 RiboMAX Express RNAi System (Promega). The unrelated gene coding for green fluorescent protein (GFP) dsRNA was synthesized from the L3790 plasmid following the same protocol and used as a negative control. L3790 was a gift from Andrew Fire (Addgene plasmid # 1596; http://n2t.net/addgene:1596, accessed on 12 October 2020; RRID: Addgene1596). The SUB dsRNA was used as a positive control as previously described [2,42]. The dsRNA was quantified by spectrophotometry and maintained at −80 °C. Unfed *I. ricinus* adult female ticks (*n* = 80 per group) were injected with approximately 0.5 μL of dsRNA (7 × 10^11^–8 × 10^11^ molecules/μL) in the lower right quadrant of the ventral surface of the exoskeleton [53]. The injections were made using a 10 μL syringe with a 20 mm, 34-gauge needle (Hamilton, Bonaduz, Switzerland). After dsRNA injection, female ticks were placed in different feeding units with the same number of *I. ricinus* males (20 females and 20 males per feeding unit) for artificial feeding. Feeding units were made and set up as previously described by Krull et al. [52]. Bovine blood used was collected from cattle and directly supplemented with heparin (20 IU/mL) (Ratiopharm, Ulm, Germany). Blood was immediately refrigerated and stored at 4 °C for up to one week. The phagostimulant adenosine triphosphate (ATP, Carl Roth) (51 mg/mL), glucose (20.5%), Vitamin B prepared following the recipe of Duron et al. [54] and the broad-spectrum antibiotic gentamycin (Roth; 5 μg/mL) were added just before each use. Feeding units were placed in a water bath at 37 °C and ticks were fed as previously described [52]. Data on the number of feeding females were recorded twice a day and engorged females that detached were weighed and stored individually in glass vials with pierced lids that were kept in a desiccator at approximately 90% relative humidity at RT. Engorgement and egg batch weights were also recorded. Values of *sub* and *importin-α* RNAi tick groups were compared with controls by one-way ANOVA test with post-hoc Tukey HSD (https://astatsa.com/OneWay_Anova_with_TukeyHSD/, accessed on 12 October 2020; *p* < 0.05).

### 3.7. Corroboration of Sub and Importin-α Silencing by qRT-PCR in I. Ricinus Organs

After three days of artificial feeding, six fed females per group were selected for RNA extraction. Dissection of fed females was performed to separate the five major organs: salivary glands, fat body, Malpighian tubules, ovary and midgut (2 ticks per biological replicate, 3 biological replicates per group). Organs were placed in 100 μL of TRIzol Reagent (Invitrogen) and total RNA was extracted and gDNA removed using the Direct-Zol RNA Miniprep Plus Kit (Zymo Research Europe GmbH) following the manufacturer’s recommendations. cDNAs of each sample were reverse-transcribed using the ProtoScript II First Strand cDNA Synthesis Kit (New England Biolabs Inc., Ipswich, MA, USA). Gene knockdown levels after *sub* and *importin-α* RNAi were assessed by qPCR on cDNA samples using gene-specific oligonucleotide primers whose targets differ from those of the dsRNAs (*sub*, F: 5′ CAGAGGAGATAGCGGCCAACATT 3′, R: 5′ GGCTCTCGCGCTCCTTCATCATG 3′; *importin-α*, F: 5′ TGGGCGCTCTCCAATCTTTG 3′, R: 5′ TCACTGCCTGGATCTTGTCG 3′), the Luna Universal qPCR Master Mix and the CFX96 Touch Real-Time PCR Detection System (BioRad). A dissociation curve was run at the end of the reaction to ensure that only one amplicon was formed, and the amplicons denatured consistently in the same temperature range for every sample. The cDNA levels were normalized against the tick *elongation factor* (*elf*) using the genNorm Delta-Delta-Ct (ddCt) method (Ayllón et al., 2013). Cycle threshold (Ct) values for normalized *sub* and *importin-α* dsRNA-treated samples were compared with those of the GFP control samples to determine levels of gene knockdown.

### 3.8. Co-Regulation Assessment of Sub and Importin-α

The cDNAs obtained above (see *2.6 Gene knockdown by RNAi in ticks*) were used to assess the *sub* mRNA levels after *importin-α* knockdown and the *importin-α* mRNA levels when *sub* was knocked down. The Ct values were normalized against the tick *elf* using the genNorm Delta-Delta-Ct (ddCt) method as described above. Normalized *sub* and *importin-α* Ct values were compared to those of the GFP control samples to determine their concentration in the absence of the corresponding interacting protein.

### 3.9. Histone Microarray Analysis

EpiTriton Histone Peptide Arrays were used following the manufacturer’s recommendations (Epicypher, Inc., Durham, NC, USA) with recombinant *I. scapularis* SUB and *Anaplasma phagocytophilum* heat shock protein 70 (HSP70) as a negative control. Both recombinant proteins, SUB and HSP70, were produced in *E. coli* as previously described [55]. Proteins were applied to the histone array diluted in array buffer (phosphate-buffered saline PBS, 5% BSA (*w*/*v*), 0.1% Tween-20) at 2 μM concentration and incubated overnight at 4 °C. The next day, arrays were washed with array buffer and incubated with a protein-specific 1:500 dilution of primary rabbit polyclonal antibodies for SUB [56] and HSP70 [55] for 2.5 h at RT. Slides were washed with PBS and probed with a fluorescently labelled Alexa Fluor 635 goat anti-rabbit IgG secondary antibody (A31576; Invitrogen). Microarrays were scanned and analyzed using a Genepix Personal 4100 A microarray scanner (Molecular Devices, LLC., San Jose, CA, USA) for measuring fluorescence in relative fluorescence units (RFU) (Data S1). Fluorescence was normalized against control empty spots (average of 87 technical replicates) and the values compared between SUB and HSP70 by Student’s t-test (*p* < 0.05) and One-Way ANOVA (https://goodcalculators.com/one-way-anova-calculator/, accessed on 12 October 2020; *p* < 0.05) tests (*n* = 2 technical replicates).

### 3.10. Corroboration of SUB-Histone 4 (H4) K-Butyrylated (H4but) Peptide Interaction by Protein Pull-Down and Western Blot Analysis

The recombinant *I. scapularis* SUB was produced as described above and used for interaction with the synthetic peptide of human H4 K5,8,12 but (amino acids 1–23, Ac-SGRGK (but) 5GGK (but) 8GLGK (but) 12GGAKRHRKVLR-Peg (Biot), butyrylated at lysines 5, 8 and 12). Dynabeads MyOne Streptavidin T1 (Invitrogen) was used following the manufacturer’s instructions. As a negative control, the same procedure was carried out but without incubation with the H4but peptide. The recombinant SUB was used as a positive control. Eluted protein-H4 complexes and positive and negative controls were separated by electrophoresis in a 12% SDS polyacrylamide precast gel (ClearPage) and transferred to a nitrocellulose blotting membrane (GE Healthcare Life Sciences). The membrane was blocked with 3% BSA in TBS (150 mM NaCl, 50 mM Tris-HCl, pH 7.5) for 2 h at RT and washed four times with TBS-0.05% Tween 20. SUB-specific primary antibodies diluted in TBS were used for SUB detection and incubated with membrane overnight at 4 °C. Goat anti-rabbit IgG (whole molecule) peroxidase antibodies (dilution 1:1000; Sigma-Aldrich) diluted in TBS with 3% BSA were used as secondary antibodies and incubated with the membrane for 2 h at RT. The membrane was finally washed five times with TBS-0.05% Tween 20, and immunoreactive proteins were visualized with chemiluminescence by incubating the membrane for 2 min with Pierce ECL Western blotting substrate (Thermo Fisher Scientific) and with TMB Stabilized Substrate for Horseradish Peroxidase (Promega).

## 4. Conclusions

The characterization of the interactome of a co-factor regulatory protein such as SUB is crucial to understand its function in the cell regulome. The physical interaction of SUB with Importin-α suggested one pathway of entrance into the nucleus for SUB, but the results also suggested that importin-α mediated entry may be not the only mechanism. Moreover, SUB has evolved to exert several functions and to interact with various other proteins in these capacities. The interaction of SUB with histone H4 also suggested new mechanisms underlying a role for this protein in chromatin remodeling and the regulation of gene expression. The expression of *importin-*α has been described to be regulated by miRNAs in humans [38]. For example, miR-223 was shown to inhibit the NF-kB signaling pathway by targeting Importins α4 or α5 and reducing its expression [57]. SUB/AKR are involved in the Relish/NF-kB gene regulation pathway playing an important role in several cellular functions, such as immune response to bacterial infection [21,58]. Pathogens like *Anaplasma phagocytophilum* are able to manipulate the tick miRNA profile to facilitate infection by upregulating isc-mir-79 in *I. scapularis* tick cells [59], but the functional implications for the regulation of *importin-*α are still unknown. In light of our results, along with the previously described functions of Importin-α in gene regulation and epigenetic modifications, further studies will be required to unravel the significance of the SUB-Importin-α interaction in different biological contexts. Moreover, the characterization of the SUB interactome and its interacting proteins such as Importin-α opens the possible identification of new targets for interventions to control tick infestation and pathogen infection that would contribute to vaccine development.

## Data Availability

All the data are included within the article and its additional files. The tick cDNA library was previously published [49] and will be made available upon request.

## Supplementary Materials

The following are available online at https://www.mdpi.com/article/10.3390/pathogens10040457/s1, Data S1: Microarray analysis of AKR2-Histone interactions.
